# The IRIS Registry: A Novel Approach to Clinical Registry Development in Ophthalmology

**DOI:** 10.1016/j.mcpdig.2023.10.003

**Published:** 2023-11-18

**Authors:** John C. Lin, Leslie Hyman, Ingrid U. Scott

**Affiliations:** a Department of Ophthalmology, Perelman School of Medicine, University of Pennsylvania, Philadelphia, PA; b Vickie and Jack Farber Vision Research Center, Wills Eye Hospital, and Department of Ophthalmology, Sidney Kimmel Medical College, Thomas Jefferson University, Philadelphia, PA; c Departments of Ophthalmology and Public Health Sciences, Penn State College of Medicine, Hershey, PA

Mandated registries offer rich, longitudinal data to support clinical research and health care quality improvement.^1^ However, such databases can be difficult to establish, largely because they require physician adherence to governmental and organizational regulations and impose considerable expenses on clinicians. Robust, voluntary clinical registries can also be developed by physician organizations without the need for governmental intervention, as evidenced by the American Academy of Ophthalmology (AAO) Intelligent Research in Sight (IRIS) Registry.^2^

With over 483 million visits and 78.6 million unique patients as of January 1, 2023, the IRIS Registry has become the largest single specialty clinical registry in the world.^2^ Moreover, the registry includes data from ∼70% of practicing ophthalmologists in the United States (US).^3^ Originally created to provide ophthalmologists with quality metrics,^3^^,^^4^ IRIS Registry data demonstrated that performance rates for aggregate quality measures such as adverse events and clinical outcomes improved considerably in the registry’s first 3 years.^4^ Its additional value for research was recognized soon thereafter, with over 70 studies published using IRIS data on topics ranging from relatively rare conditions like uveal melanoma to practice patterns for common procedures such as cataract surgeries.^2^ The Centers for Disease Control and Prevention has used IRIS data to calculate the prevalence of ophthalmic conditions and services.^5^ The registry has proven to be invaluable for ophthalmic research into care delivery, health disparities, and clinical outcomes, providing abundant data for additional analyses.^1^

The success of the IRIS Registry is rooted in many factors. First, the registry helps practices meet the requirements of federal quality reporting programs which, using financial incentives, encourage clinicians to report quality data.^3^^,^^4^ Second, the registry interfaces seamlessly with common electronic health record (EHR) systems.^4^ Third, the registry assures ophthalmologists of the privacy, confidentiality, and security of submitted data.^4^ Of importance, the IRIS Registry calculates quality data for practices in comparison to peers, identifying areas of improvement for clinicians.^4^

Although the IRIS Registry is limited by its voluntary sample and reliance on EHRs, its inclusion of 70% of practicing US ophthalmologists highlights its feasibility and use. The IRIS Registry is operated by the AAO in partnership with the for-profit corporation Verana Health, which has connections to leaders in ophthalmology, some of whom benefit financially from the IRIS Registry. However, mandated registries may also face issues with noncompliance and similarly rely on EHR data and commercial vendors. Overall, the development of the IRIS Registry provides valuable lessons for all specialties similarly seeking to create and potentially link registries. Limitations of the IRIS Registry can be mitigated by incentivizing high-quality EHR maintenance and transparently addressing potential conflicts of interests. Future advances may involve the inclusion of more systemic data, comprehensive eye examination findings, and additional clinical images. With more high-quality and comprehensive data available, physicians will have more tools with which to improve their patient care.

## Potential Competing Interests

The authors report no competing interests.
